# Supplementary material for: Influenza Vaccine Effectiveness in Preventing Laboratory-Confirmed Influenza Cases and Hospitalizations in Navarre, Spain, 2022–2023
Source: Vaccines (Basel). 2023 Sep 12;11(9):1478. doi: 10.3390/vaccines11091478 (PMC10534462; doi:10.3390/vaccines11091478)
Supplement: Supplementary file 1 [file vaccines-11-01478-s001.zip › vaccines-2578661-supplementary.pdf]

Supplementary material

Supplementary Table S1. Distribution of influenza virus (sub-)types by healthcare setting

Subtype influenza virus	All samples n (%)	Primary healthcare n (%)	Hospital n (%)
**Total**	571 (100%)	222 (100%)	349 (100%)
A(H3N2)	348 (61%)	123 (55%)	225 (64%)
A(H1N1)	85 (15%)	24 (11%)	61 (17%)
B	95 (17%)	68 (31%)	27 (8%)
A not subtyped	43 (8%)	7 (3%)	36 (10%)

Comparison of (sub-)type influenza viruses between primary healthcare and hospital patients using the χ^2 test, P<0.001

Supplementary Figure S1. Phylogenetic tree representing all characterized A(H3N2) influenza viruses from Navarre, Spain, 2022-2023 influenza season

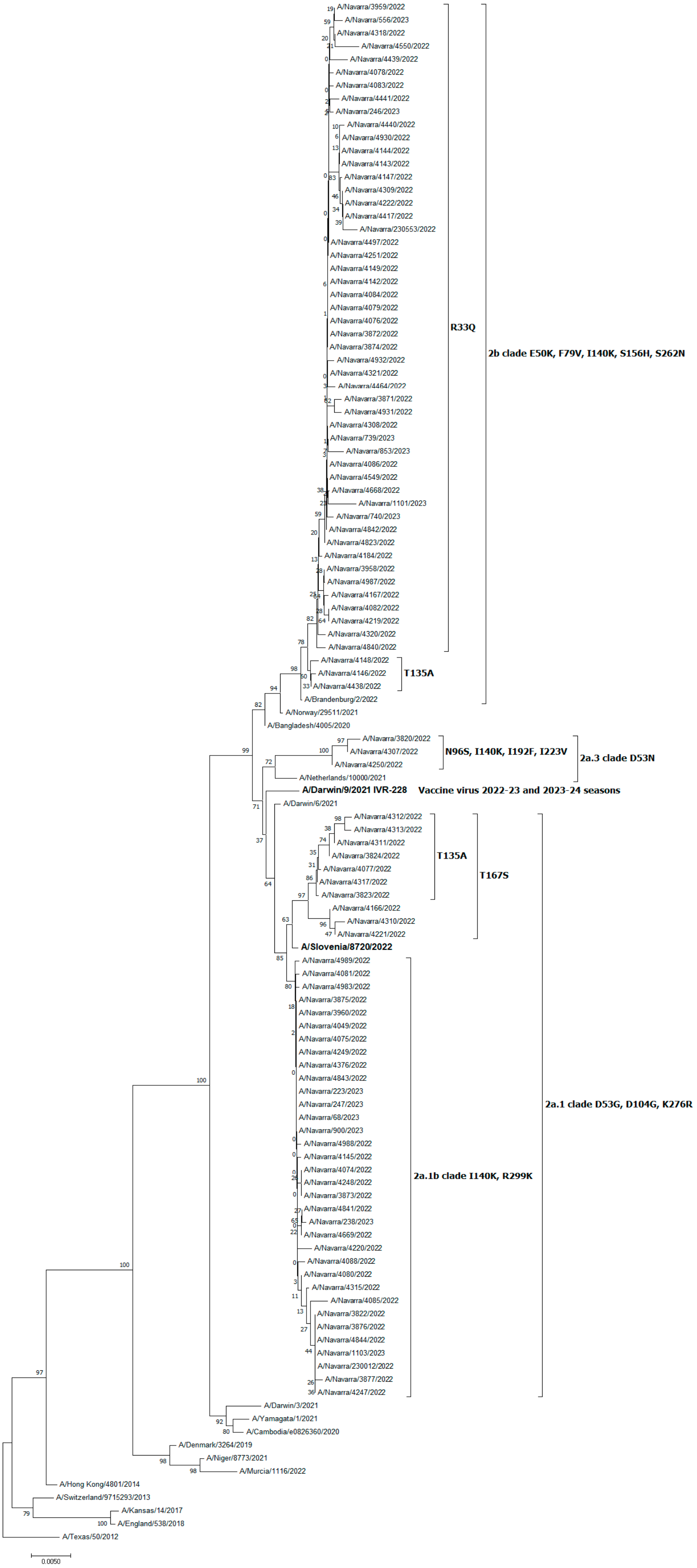

Supplementary Figure S2. Phylogenetic tree representing all characterized B Victoria influenza viruses from Navarre, Spain, 2022-2023 influenza season

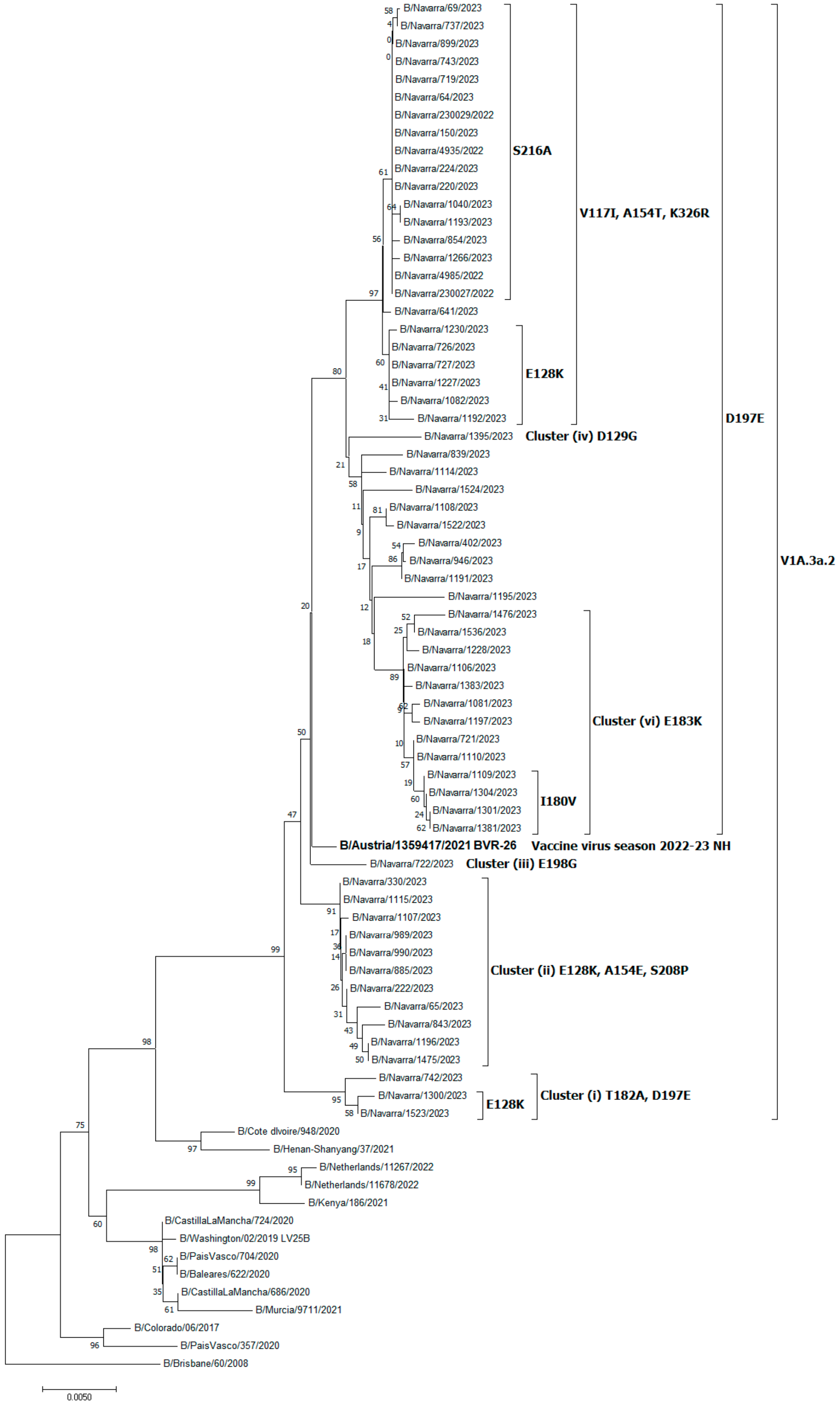

Supplementary Table S2. Effect of influenza vaccination status in the current and three prior seasons in preventing laboratory-confirmed influenza

	Cases / controls	Crude vaccine effectiveness, % (95% CI)	Adjusted vaccine effectiveness,% (95% CI) [a]	*P value*
**All patients**				
Never vaccinated	252/678	1	1	
Vaccination in prior seasons only	114/519	41 (24 to 54)	6 (–27 to 30)	0.692
Current season vaccination	205/1553	64 (56 to 71)	36 (16 to 51)	0.001
**All outpatients**				
Never vaccinated	165/187	1	1	
Vaccination in prior seasons only	31/51	31 (–13 to 58)	8 (–58 to 46)	0.765
Current season vaccination	26/98	70 (51 to 81)	49 (8 to 71)	0.025
**All inpatients**				
Never vaccinated	87/491	1	1	
Vaccination in prior seasons only	83/468	0 (–39 to 28)	0 (–45 to 31)	0.999
Current season vaccination	179/1455	31 (8 to 47)	24 (–6 to 45)	0.109
**Aged 9 to 64 years**				
Never vaccinated	209/461	1	1	
Vaccination in prior seasons only	47/135	23 (–11 to 47)	–3 (–56 to 32)	0.880
Current season vaccination	35/186	58 (38 to 72)	31 (–8 to 55)	0.105
**Aged ≥65 years**				
Never vaccinated	43/217	1	1	
Vaccination in prior seasons only	67/384	12 (–34 to 42)	16 (–32 to 47)	0.441
Current season vaccination	170/1367	37 (10 to 56)	33 (4 to 55)	0.030
**Target population**				
Never vaccinated	108/437	1	1	
Vaccination in prior seasons only	95/478	20 (–9 to 41)	0 (–41 to 29)	0.985
Current season vaccination	195/1528	48 (33 to 60)	35 (13 to 52)	0.004
**A/H3N2 subtype** [b]				
Never vaccinated	131/522	1	1	
Vaccination in prior seasons only	91/466	22 (–5 to 42)	–11 (–58 to 22)	0.568
Current season vaccination	125/1088	54 (40 to 65)	24 (–7 to 46)	0.115
**A/H1N1 subtype** [c]				
Never vaccinated	25/440	1	1	
Vaccination in prior seasons only	4/167	58 (–23 to 85)	37 (–92 to 80)	0.412
Current season vaccination	45/1140	30 (–15 to 58)	–24 (–143 to 37)	0.537
**B type** [d]				
Never vaccinated	75/344	1	1	
Vaccination in prior seasons only	8/103	64 (24 to 83)	60 (0 to 84)	0.050
Current season vaccination	9/958	96 (91 to 98)	88 (69 to 95)	<0.001

Abbreviations: CI, confidence interval.

[a] Vaccine effectiveness adjusted by sex, age groups (9–24, 25–44, 45–64, 65–84 and ≥85 years), presence of major chronic conditions, healthcare setting (primary healthcare or hospital), and month of sample collection.

[b] A/H3N2 analysis includes months between October 2022 to February 2023.

[c] A/H1N1 analysis includes months between December 2022 to March 2023.

[d] B analysis includes months between January to April 2023.

Supplementary Table S3. Sensitivity analysis of influenza vaccine effectiveness in preventing laboratory-confirmed influenza, including COVID-19 confirmed cases in the control group

	Cases / controls	Crude vaccine effectiveness, % (95% CI)	Adjusted vaccine effectiveness,% (95% CI) [a]	*P value*
**All patients**				
Unvaccinated	366/1375	1	1	
Vaccinated	205/1760	56 (47 to 64)	33 (15 to 47)	0.001
**All outpatients**				
Unvaccinated	196/288	1	1	
Vaccinated	26/119	68 (49 to 80)	50 (13 to 71)	0.014
**All inpatients**				
Unvaccinated	170/1087	1	1	
Vaccinated	179/1641	30 (13 to 44)	21 (–4 to 40)	0.096
**Aged 9 to 64 years**				
Unvaccinated	256/680	1	1	
Vaccinated	35/213	56 (36 to 70)	32 (–3 to 56)	0.069
**Aged ≥65 years**				
Unvaccinated	110/695	1	1	
Vaccinated	170/1547	31 (10 to 46)	24 (–3 to 44)	0.073
**Target population**				
Unvaccinated	203/1052	1	1	
Vaccinated	195/1731	42 (28 to 53)	33 (14 to 48)	0.002
**A/H3N2 subtype** [b]				
Unvaccinated	222/1128	1	1	
Vaccinated	125/1215	48 (34 to 59)	25 (–1 to 44)	0.053
**A/H1N1 subtype** [c]				
Unvaccinated	29/706	1	1	
Vaccinated	45/1296	15 (–36 to 47)	–37 (–156 to 27)	0.327
**B type** [d]				
Unvaccinated	83/511	1	1	
Vaccinated	9/1090	95 (90 to 97)	84 (62 to 93)	<0.001

Abbreviations: CI, confidence interval.

[a] Vaccine effectiveness adjusted by sex, age groups (9–24, 25–44, 45–64, 65–84 and ≥85 years), presence of major chronic conditions, healthcare setting (primary healthcare or hospital), and month of sample collection.

[b] A/H3N2 analysis includes months between October 2022 to February 2023.

[c] A/H1N1 analysis includes months between December 2022 to March 2023.

[d] B analysis includes months between January to April 2023.
